# Barriers and facilitators associated with the use of mental health services among immigrant students in high-income countries: a scoping review protocol

**DOI:** 10.1186/s13643-022-01896-6

**Published:** 2022-02-06

**Authors:** Christelle Dombou, Olumuyiwa Omonaiye, Sarah Fraser, Jude Mary Cénat, Sanni Yaya

**Affiliations:** 1 Interdisciplinary School of Health Sciences, University of Ottawa, Ottawa, Ontario Canada; 2 School of Nursing and Midwifery, Centre for Quality and Patient Safety Research, Institute for Health Transformation, Deakin University, Burwood, Australia; 3 Centre for Nursing and Midwifery Research, James Cook University, Townsville, Queensland Australia; 4 School of Psychology, University of Ottawa, Ottawa, Ontario Canada; 5 School of International Development and Global Studies, University of Ottawa, Ottawa, Ontario Canada; 6 The George Institute for Global Health, Imperial College London, London, UK

**Keywords:** Mental health, Immigrant students, High-income countries, Barriers, Facilitators

## Abstract

**Background:**

While the mental health of immigrants is a growing issue that is attracting increasing interest from researchers, the same cannot be said for the mental health of immigrant students especially for international students. Indeed, the mental health of immigrant students and their use of mental health services are still poorly documented despite the significant increase in the number of these students in many high-income countries. This scoping review aims to providing an overview and exploring gaps in existing research regarding access to mental health care among immigrant students by identifying barriers and facilitators associated with the use of mental health services in high-income countries.

**Methods:**

With the help of a professional librarian, we will develop a search strategy including several keywords such as mental health, mental illness, immigrant, students, immigrant students, or international students and access to care or use of mental health services. The following electronic databases will be searched (from their inception onwards): MEDLINE, APA PsycINFO, CINAHL, Web of Science Core Collection, Education Source, and Embase. Studies addressing access to and use of mental health care conducted on immigrant students (adolescent and above) in high-income countries will be included. Two reviewers will independently screen all citations, full-text articles, and abstract data. A narrative summary of findings will be conducted. Data analysis will involve quantitative (e.g., frequencies) and qualitative (e.g., content and thematic analysis) methods.

**Discussion:**

The purpose of this scoping review is to better map the literature on the mental health of immigrant students and their use of mental health care services. In doing so, we aim to identify barriers and facilitators to access and use of mental health care. Identifying barriers and facilitators of mental health services by immigrant students will support the development of appropriate interventions that can help improve access and use of mental health services by immigrant students in high-income countries.

**Systematic review registration:**

Open Science Framework (osf.io/a2rk6)

**Supplementary Information:**

The online version contains supplementary material available at 10.1186/s13643-022-01896-6.

## Background

Research on immigration and mental health is attracting increasing global interest [1–3]. Immigration to high-income countries has been on the rise in recent decades and this has multiple causes and consequences on the host countries. For example, since the Confederation in 1867, Canada has welcomed more than 17 millions of immigrants from different cultures around the world [4]. At the time of the last census in 2016, there were approximately 7,540,830 foreign-born people in Canada, representing 21.9% of the Canadian population [5]. For their part, international students arrive in the tens of thousands at Canadian institutions each year. According to Canadian Bureau for International Education, international students are a vital element in the internationalization of Canadian institutions and Canadian society [6].

### Factors affecting mental health of immigrant

Since mental health is a guarantee of well-being and the proper functioning of individuals and society [7], governments are aware of its importance for a healthy and a productive society. Numerous studies have shown the association between migration and mental health [8–10]. Upon arrival, immigrants often have generally better health status than natives including mental health: the so-called healthy immigrant effect [11, 12]. This health status tends to deteriorate over time [13–15] because the migratory journey brings its share of changes, stress, adaptation, trials, and unforeseen events. Deterioration in mental health is known to be multifactorial, as several factors pre-, during and post-immigration can have an impact on mental health [8]. This deterioration is associated with a series of changes that affects the physical and psychological well-being of immigrants [10, 16, 17]. Several studies have demonstrated that some post-migration factors such as settlement and integration to their new environment, unemployment, education, poverty in the USA [18]; racism and discrimination in France [19]; and also, the loss and lack of family or social support when immigrating in Canada [20] had negative effects on immigrants’ health. Additionally, acculturation stress can also play a role in the amplification of mental illness such as depressive disorders and psychological distress for Latino and Asian refugees and immigrants in USA [21]. Hence, given the aforementioned, immigrants are at risk of developing mental illness.

### Barriers of the use of mental health services

In line with the World Health Organization (WHO) International Classification of functioning, disability, and health (ICF; 2001), contextual factors (environmental and personal) can include barriers and facilitators that can either diminish or improve one’s functioning and health [22]. One of the main factors associated to immigrants’ mental illness is the barriers that prevent or minimize the use of mental health services [20]. First, the cultural differences that immigrants face in their host country may constitute a barrier to health care utilization [23]. According to a Canadian study, culture can lead to ignorance, withdrawal, and stereotyping about mental problems. This may lead some immigrants to avoid seeking specialized care for fear of discrimination and stigmatization [20]. Second, the language can also constitute a barrier to mental health care utilization. According to a US study among Chinese and Latino immigrants with psychiatric disorders, limited English language proficiency was a barrier to the use of mental health services among these immigrants [24]. In addition, a study conducted in providing an overview of reported barriers to accessing mental health care among culturally and linguistically diverse immigrant women in Australia [25]. In the aforementioned study, the authors noted that in addition to language and logistical barriers (such as lack of information and knowledge of the care system, lack of finances, lack of private health insurance, transportation difficulties, long waiting lists, and delays in seeking professional care), there were also reportedly several cultural disagreements between mental health care providers and these women. Some women felt disrespected during consultations and would have preferences for alternative interventions such as solving their problems alone or having help from family or friends. Despite the existence of government-subsidized integration programs in high-income countries such as Canada, the USA, and Australia, evidence shows that newly settled immigrants underutilize the existing mental health services and remain affected by mental illness.

### Case of immigrant/foreign students: statistics and particular problems

According to UNESCO, there were more than 5.3 million higher education students abroad in 2017 [26]. According to the 2018 report of the Canadian Bureau for International Education, they were already nearly 494,525 international students in Canada in 2017 [6], and in the 2020 Open Doors Report on International Educational Exchange published by the Institute of International Education (IIE), the USA welcomed more than one million international students during the 2019/2020 academic year [27]. In its 2019 report, the Atlas Project classifies the countries in the world that received the most international students in 2019. Among the top 5 are the USA with 1,095,299, the UK with 496,570, China with 492,185, Canada with 435,415, and Australia with 420 501 students [28].

In general, international students present the same mental health problems as other categories of immigrants: they are generally in good health upon arrival, but their health deteriorates once they are in their host country. According to Michaud, this deterioration in health can result from social factors and determinants such as lack or poor social and family support, non-employability or under employability or poverty, and acculturation and other stresses [29]. In addition, international students face many other additional challenges and barriers that can make them psychologically and socially vulnerable [29]. These obstacles may interfere with the different aspects of the international student experience (socio-cultural, academic, administrative and financial, personal) at different times (preparation, arrival, return to the country of origin) [30].

High-income countries generally have government-subsidized integration programs to help newly settled immigrants to integrate in their new country. However, it is important to note that immigrants on temporary visas, such as international students, are generally not targeted or affected by these programs. This makes them more vulnerable than other categories of immigrants, especially since they are in situations that are specific to them [31]. According to the Facilitation Consortium on Persistence and Success in Higher Education (CAPRES in French) in Québec, immigrant students are more at risk of developing mental illness since they encounter many obstacles that can increase their vulnerability compared to other categories of immigrants [32]. These barriers are culture shock; the social network to be (re)built; adaptation to teaching and learning; language barriers; racism and prejudice [31].

Previous studies have addressed barriers to health service use by international students. However, the literature remains limited and less clear on general predictors of mental health service use among this population. Our topic is fairly new, and the subject is broad at the same time. The objective of this scoping review is to provide an overview of available research in this area and to identify existing gaps in what is known about the mental health of immigrant students and their use of mental health services. Hence, we want to identify all the different barriers and facilitators to mental health service use among immigrant students based on all relevant articles, regardless of their designs [33]. We also want to categorize these barriers and facilitators to better adapt them to specific contexts and thus, specify the recommendations that can be drawn up as a result of this research. A scoping review was chosen because it is more suitable as a method to achieve our research objective—scoping reviews are conducted to map the literature on a particular area of research that has not been previously and methodically reviewed, therefore providing an opportunity to identify important concepts, gaps in research and key sources and types of evidence, to inform research, practice, and policymaking [33, 34]. Thus, this is the first review, to our knowledge, that focuses specifically on immigrant students and aims to identify barriers and facilitators to the use of mental health services by immigrant students in high-income countries.

## Methods

This study protocol has been registered within the Open Science Framework (pre-registration number: [osf.io/a2rk6]) and is being reported in accordance with the reporting guidance provided in the Preferred Reporting Items for Systematic Reviews and Meta-Analyses Protocols (PRISMA-P) statement [35] (see checklist in Additional file 1). The planned review will be reported according to the PRISMA Extension for scoping review (PRISMA-ScR) [36]. This scoping review protocol will be conducted by using Joanna Briggs Institute (JBI) guidelines for conducting scoping reviews to ensure systematic and repeatable work [37] and will follow the five stages included when conducting a scoping review as outlined by Arksey and O’Malley [33].

### Protocol design

#### Stage 1: Identification of the research question and the objectives

The purpose of this Scoping Review is to provide an overview of available research in this area and to identify existing gaps on what is known about the mental health of immigrant students and their use of mental health services. The main question that will guide the inquiry, analysis, and consolidation of evidence in this scoping review is as follows: What are barriers to and facilitators of the utilization of mental health services by immigrant students in High-Income countries?

We used Cooke, Smith & Booth’s SPIDER tool to formulate our research question, as it best suited our research objectives [38]. Where *S* (Sample) are immigrant students; *P* of *I* (Phenomenon of Interest) are mental health barriers and facilitators; *D* (Design) include all designs (questionnaires, survey, interviews, focus group, case study, or observational study); *E* (Evaluation/Assessment) is students’ experience in accessing care (e.g., positive= facilitators; negative = barriers) and *R* (Research type) are quantitative, qualitative or mixed methods.

#### Stage 2: Identifying relevant studies

##### Data sources

With the help of a professional librarian, we will develop a search strategy including several keywords such as mental health, mental illness, immigrant, students, immigrant students, or international students and access to care or use of mental health services. From these keywords, we will develop MeSH terms for database searches. Six databases will be selected for our searches: MEDLINE, APA PsyInfo, Education Source, CINAHL, Web of Science Core Collection, and EMBASE. The search for articles will be carried out on these databases from their inception onwards. Two reviewers will independently screen all articles in Covidence software. A draft search strategy for MEDLINE is available in Additional file 2.

A bibliographic software, Zotero, will be used to store, organize, and manage all references [39]. Covidence will be used to manage the title/abstract and full-text screening phases.

##### Eligibility criteria

As with all review studies, we have established inclusion and exclusion criteria for this systematic scoping review. In general, only studies published in English on immigrant students who were adolescents or older, studying in a high-income country at the time of the study, and who have used (or intend to use) mental health care will be included in this review. In contrast, we will exclude all studies that do not meet these criteria (i.e., those that did not include immigrant students, were under 10 years of age, did not address mental health care utilization, were conducted in low-income countries, and were published in a language other than English). Since knowledge on the subject is still limited, date will not be a criterion for inclusion/exclusion. In other words, we will include all studies that meet the inclusion criteria, regardless of when the study was conducted or published. In more detail:

- *Population*: Eligible studies will include any immigrant/international students and will be defined as follows all persons who are at least 10 years of age or older and who are studying at an educational institution recognized by their host country where they are living at the time of the study. This age range adolescence (10 years and older) was chosen based on the definition of the World Health Organization (WHO) [40].
- *Concepts*: Studies that identify strategies, barriers, facilitators, or outcomes and contextual factors in the use of mental health services will be included. Papers refining or developing theory, conceptual models, and frameworks will be excluded, unless they also describe barriers, facilitators, and strategies or outcomes (e.g., attitudes, beliefs, knowledge, benefits, and unintended consequences).
- *Study designs*: All study designs using qualitative or quantitative methods will be eligible for inclusion, except for case reports. Specifically, we will include experimental (such as randomized controlled trials and non-randomized clinical trials), quasi-experimental (interrupted time series, controlled before-after studies), observational (cohort, case-control, cross-sectional, case series), and qualitative studies (interviews, open-ended questionnaires, focus groups). We will exclude systematic reviews or other reviews.
- *Context*: Studies conducted in high-income countries will be considered for inclusion.
- *Other*: Only papers written in English will be considered for inclusion.

##### Inclusion criteria

- Articles addressing access to and use of mental health care
- Articles about immigrant/international students
- Participants in the study are adolescents and above
- Original language is English
- High-income countries

##### Exclusion criteria

- Do not address access to and use of mental health care
- Not about immigrant/international students
- Studies involving on children (less than 10 years old)
- Language other than English
- Systematic review or other reviews
- Any commentaries, editorials, or opinion pieces
- Low-income countries

### Stage 3: Study selection

Two independent reviewers will review all articles in Covidence according to the above-mentioned inclusion/exclusion criteria. To increase consistency among reviewers, they will screen a sample of 20 publications, discuss the results and amend the screening and data extraction manual before beginning the screening for this review. Two reviewers will be working in pairs sequentially to evaluate the titles, abstracts, and then full text of all publications identified by the searches for potentially relevant publications. Disagreements on study selection and data extraction are expected to be solved by consensus and discussion with the third reviewer if needed. This will be done as follows: after importing the articles into Covidence, this software will automatically eliminate all duplicates (studies identified more than once by the search engines) and the two reviewers will start screening the articles. In a first step (first sort), the titles, abstracts, and summaries (if any) will be examined and all studies that clearly do not meet the inclusion criteria will be excluded. Each study will be classified as “included” or “excluded” to identify relevant literature and to exclude irrelevant literature. If there is any doubt about the relevance of a publication, the reviewer may leave it for further evaluation to include or exclude it. In a second phase, all full-text articles that meet the inclusion criteria will be evaluated in more detail. All full-text articles will be carefully reviewed and those that do not meet the listed inclusion criteria will be excluded. At the end, we will only extract and analyze articles that met our inclusion criteria. A PRISMA diagram that shows the complete selection of articles for this journal will be presented to understand the process of what has been done.

### Stage 4: Data extraction and charting the data

Data from articles that meet our inclusion criteria will be extracted into an Excel spreadsheet developed from the adaptation of the manual (data extraction) the Joanna Briggs Institute [14]. This data extraction spreadsheet will be developed by a first reviewer who will be shared with a second reviewer (and a third reviewer if needed) to ensure that it will be reasonably interpreted and will capture all relevant data from different study designs to meet the objectives of the study. This Excel sheet will be developed and well-tailored to record key information from the studies to be analyzed. It will include authors, year of study, objective/purpose, geographic area (e.g., country), study population (e.g., age and gender of participants, degree on education), sample size, study design, results (e.g., barriers/facilitators) and key findings related to question and objective of this review. This will be consistent with the Joanna Briggs Institute Manual [14], and scoping reviews on health-related topics such as the scoping review protocol on *Barriers, facilitators, strategies and outcomes to engaging policymakers, healthcare managers and policy analysts in knowledge synthesis: a scoping review protocol* by Tricco et al. [41].

### Stage 5: Collating, summarizing, and reporting the data

We will use a narrative review approach to collect and summarize the characteristics of each article (e.g., country of origin, type, and nature of the study, group of the age of participants, the date, and results) and present them without attempting to assess the certainty of the results. We will involve quantitative (e.g., frequencies) and qualitative (e.g., content and thematic analysis) methods to present the data collected in different forms (tables, figures, texts). Synthesis of the results including socio-demographic characteristics factors and more elements of the participants will help in the analysis and consistency of this review. Graphs can also be used to present the results in a clearer and more understandable way.

#### Quality assessment and risk of bias

Appraisal for quality assessment or risk of bias will not be performed because this is a scoping review. This is consistent with the Joanna Briggs Institute Manual [14] and scoping reviews on health-related topics such as the scoping review protocol on *Barriers, facilitators, strategies and outcomes to engaging policymakers, healthcare managers and policy analysts in knowledge synthesis: a scoping review protocol* by Tricco et al. which does not evaluate the risk of bias [41]. In this review, articles will be reviewed by at least two independent reviewers. This will minimize the risk of bias during the sorting of articles. However, certain restrictions such as language and the nature of the articles chosen for the review (primary or secondary) may constitute risks of bias and this may be limitations for our review. Relevant articles written in languages other than English may be missed because of limiting searches to only articles published in the English language. For example, we know that according to the Project Atlas [28], China was one of the top 5 high-income countries to welcome the most foreign students in 2019. However, the main language in China is Mandarin. So, by considering only articles written in English, our search is considerably restricted, and therefore, we would probably lose several relevant articles written in Mandarin and other Chinese languages.

Also, this review can only include primary studies by its nature. However, some secondary studies, such as systematic reviews, could include relevant articles on our topic. Since we are not going to consider these types of studies, we may then miss these articles and not consider them. This could have an impact on the objectivity of our final analysis. Especially since we anticipate that it will be difficult to analyze the studies by considering and taking into account the context of each one. It is also for this reason that we will only consider high-income countries in our review in order to minimize the differences (economic, social, demographic, political, etc.) between them. However, we must keep in mind that, even though these countries have several basic similarity criteria, the population under study (immigrant students) may come from various places and contexts with characteristics that are often completely opposite to those of the host countries.

It is also important to point out that relevant articles could be eliminated in the first part of the screening (abstract and title screening) just because the keywords were not included in the title and abstract. Also, some human errors such as accidental deletion of relevant articles can happen.

## Discussion

The aim of this review is to collate, summarize, and report on study findings to identify evidence gaps and draw conclusions from the existing literature in relation to the mental health of immigrant students and their use of mental health care services in high-income countries. Thus, the rigorous and systematic nature of this review will help to identify barriers and facilitators to access and use of mental health among immigrant students in their host countries. Identifying barriers and facilitators of mental health services by immigrant students can help direct and better focus positive mental health actions for immigrant students in high-income countries where they are contributing significantly to the social and economic development of these countries. For example, international students contributed almost 38 billion dollars to the Australian economy in 2019 [42]. In Canada, international students contributed approximately $21.6 billion to the country’s GDP in 2018 [43]. According to London Economics, first-year international students enrolled in British higher education institutions had an estimated impact on the economy of £20.3 billion for the 2015–2016 academic year [44]. One practical issue or operational issue we anticipate as we conduct this scoping review will be the challenge of finding a balance between a search strategy that will not be too narrow nor too broad, and fine tuning the search during the course of the review process to make room for new vocabulary terms. In addition, since this review is part of a graduate thesis work, reviewers will be working within a compressed timeframe. Due to our time constraint, the literature search will be limited to the bibliographic databases such as Web of Science Core Collection, MEDLINE. We anticipate some limitations in this review. A potential limitation at the source of evidence level is that some data may be underreported or misreported due to the sensitive and highly stigmatizing nature of mental health issues among immigrant populations including immigrant students. Given that this a scoping review, our research question is broad in nature; hence, we anticipate that the findings from this review may likewise be broad, thus demanding extra effort and steps on the part of the reviewers to synthesize and produce practical and usable conclusions from them. If there is a need for us to amend this protocol, we would give the date of each amendment. Furthermore, we would give a detailed account and rationale for the change in this section. This research will be published in a peer-reviewed journal (the Biomedical Central Journal such as Systematic Reviews) and the results of this research will be presented in seminars, research conferences, and interactions with potential knowledge users.

## Supplementary Information

**Additional file 1.** Preferred Reporting Items for Systematic reviews and Meta-Analyses extension for Scoping Reviews (PRISMA-ScR) Checklist.

**Additional file 2.** Draft search strategy for Medline.

## Data Availability

Data sharing is not applicable to this article because no datasets were generated or analyzed for the study.

## Abbreviations

PRISMA-ScR: Preferred Reporting Items for Systematic Reviews and Meta-Analyses Extension for Scoping Reviews
WHO: World Health Organization
