# Supplementary material for: Barriers and facilitators associated with the use of mental health services among immigrant students in high-income countries: a scoping review protocol
Source: Syst Rev. 2022 Feb 6;11:22. doi: 10.1186/s13643-022-01896-6 (PMC8818184; doi:10.1186/s13643-022-01896-6)
Supplement: Supplementary file 1 — Additional file 1. Preferred Reporting Items for Systematic reviews and Meta-Analyses extension for Scoping Reviews (PRISMA-ScR) Checklist. [file 13643_2022_1896_MOESM1_ESM.docx]

**PRISMA-P (Preferred Reporting Items for Systematic review and Meta-Analysis Protocols) 2015 checklist: recommended items to address in a systematic review protocol***

| Section and topic | Item No | Checklist item |
| --- | --- | --- |
| ADMINISTRATIVE INFORMATION | | |
| Title: |  |  |
| Identification | 1a | Barriers and facilitators associated with the use of mental health services among immigrant students in high-income countries: A scoping review protocol |
| Update | 1b | Not applicable. |
| Registration | 2 | Open Science Framework (osf.io/a2rk6) |
| Authors: |  |  |
| Contact | 3a | Christelle Dombou^1^, Olumuyiwa Omonaiye^2,3^, Sarah Fraser^1^, Jude Mary Cénat^4^, Sanni Yaya^5,6^     1. Interdisciplinary School of Health Sciences, University of Ottawa, Ottawa, Ontario, Canada 2. School of Nursing and Midwifery, Centre for Quality and Patient Safety Research, Institute for Health Transformation, Deakin University, Australia 3. Centre for Nursing and Midwifery Research, James Cook University, Townsville, Queensland, Australia 4. School of Psychology, University of Ottawa, Ontario, Canada 5. School of International Development and Global Studies, University of Ottawa, Ottawa, Ontario, Canada 6. The George Institute for Global Health, Imperial College London, London, United Kingdom     Corresponding Author:  Dr. Sanni Yaya; sanni.yaya@uOttawa.ca  University of Ottawa, Ottawa, ON, Canada    CD: [cdomb015@uottawa.ca](mailto:cdomb015@uottawa.ca)  OO: [m.omonaiye@deakin.edu.au](mailto:m.omonaiye@deakin.edu.au)  SF: [Sarah.Fraser@uottawa.ca](mailto:Sarah.Fraser@uottawa.ca)  JMC: [jcenat@uottawa.ca](mailto:jcenat@uottawa.ca)  SY: [sanni.yaya@uottawa.ca](mailto:sanni.yaya@uottawa.ca) |
| Contributions | 3b | CD and SY come up with the research question and conceptualized the review approach. CD prepared the protocol manuscript and contributed to the development of the search strategy with the help of OO. OO, SF and JMC contributed to the development of the study protocol. SY had final responsibility to submit. Authors reviewed and approved the final manuscript. |
| Amendments | 4 | If there is a need for us to amend this protocol, we would give the date of each amendment. Furthermore, we would give a detailed account and rationale for the change in this section, and it will be documented here. |
| Support: |  |  |
| Sources | 5a | Not applicable |
| Sponsor | 5b | Not applicable |
| Role of sponsor or funder | 5c | Not applicable |
| INTRODUCTION | | |
| Rationale | 6 | Some studies have addressed barriers to the use of health services by international students. For example, according to the Facilitation Consortium on Persistence and Success in Higher Education (or CAPRES in French), international students in Quebec may experience these barriers: culture shock, social network (re)construction, adaptation to teaching and learning, language barriers, racism and prejudice (31). The literature remains limited and less clear on the general predictors of mental health service utilization in this population. We chose to conduct a scoping review because it is a more appropriate method to achieve our research goal, which is to provide an overview of the available research in this area and to identify existing gaps in what is known about the mental health of immigrant students and to identify all the different barriers and facilitators to mental health service utilization among immigrant students based on all relevant articles, regardless of their designs (33). We also wish to categorize these barriers and facilitators to better tailor them to specific contexts and thus, clarify recommendations that can be made as a result of this research. Scoping reviews are conducted to map the literature on a particular research area that has not been previously and methodically examined, providing an opportunity to identify important concepts, gaps in research, and key sources and types of evidence to inform research, practice, and policy development (33,34). This will be the first review, to our knowledge, that focuses specifically on immigrant students and aims to identify barriers and facilitators to the use of mental health services by immigrant students in high-income countries. |
| Objectives | 7 | Cooke, Smith & Booth's SPIDER tool was used to formulate this research question, as it better fits our research objectives (38). Each item in this tool is defined and described as follows:  **S** ("Sample" or Sample/Population) represents immigrant students i.e., any person with an immigrant background who has student status in the country where they live. Therefore, this person can be a foreign/international student, an exchange student, a refugee student or a citizen student who was not born in the country where he/she lives and studies at the time of the study.  **P of I** ("Phenomenon of Interest") is the use of mental health services by immigrant students. We will look at all types of mental health services, both within and outside the school. This refers to various consultations at the school counselling service or elsewhere, visits to psychiatrists, psychologists, psychotherapists or other mental health care professionals.  **D** ("Design") includes all designs (questionnaires, surveys, interviews, focus groups, case studies or observational studies...).  **E** ("Evaluation/Assessment") is the students' experience of using mental health services. On the one hand, we will identify any factors or elements that may prevent, deter or be a barrier for immigrant students to use mental health care (in the past, present or future). On the other hand, we want to identify all the factors and elements that may encourage or facilitate the use of mental health care by immigrant students or that may encourage them to use mental health services in the future. Thus, a positive immigrant student experience will be considered a facilitator while a negative experience will be considered a barrier.  **R** (Research type) includes quantitative, qualitative, mixed methods, experimental, quasi-experimental and observational. |
| METHODS | | |
| Eligibility criteria | 8 | **Population:** Eligible studies will include any immigrant/international students and will be defined as follows all persons who are at least 10 years of age or older and who are studying at an educational institution recognized by their host country where they are living at the time of the study. This age range adolescence (10 years and older) was chosen based on the definition of the World Health Organization (WHO) (40).  **Concepts:** Studies that identify strategies, barriers, facilitators or outcomes and contextual factors in the use of mental health services will be included. Papers refining or developing theory, conceptual models and frameworks will be excluded, unless they also describe barriers, facilitators and strategies or outcomes (eg, attitudes, beliefs, knowledge, benefits, unintended consequences).  **Study designs:** All study designs using qualitative or quantitative methods will be eligible for inclusion, except for case reports. Specifically, we will include experimental (such as randomised controlled trials, non-randomised clinical trials), quasi-experimental (interrupted time series, controlled before-after studies), observational (cohort, case-control, cross-sectional, case series) and qualitative studies (interviews, open-ended questionnaires, focus groups). We will exclude systematic reviews or other reviews.  **Context:** Studies conducted in high income countries will be considered for inclusion.  **Other:** Only papers written in English will be considered for inclusion. |
| Information sources | 9 | With the help of a professional librarian, we will develop a search strategy including several keywords such as mental health, mental illness, immigrant, students, immigrant students or international students and access to care or use of mental health services. From these keywords, we will develop MeSH terms for database searches. Six databases will be selected for our searches: MEDLINE, APA PsyInfo, Education Source, CINAHL, Web of Science Core Collection and EMBASE. The search for articles will be carried out on these databases from their inception onwards. Two reviewers will independently screen all articles in Covidence software. |
| Search strategy | 10 | **Search Strategy for Medline on Ovid MEDLINE(R) In-Process & Other Non-Indexed Citations and Ovid MEDLINE(R) «1946 to Present» (Performance date:** September 17, 2020)  1. Mental Health/  2. mental health*.ti,ab,kf.  3. exp Mental Disorders/  4. Depression/5. (mental* adj2 (disorder* or disease* or ill* or condition*)).ti,ab,kf.  6. (depress* or anxiet* or anxious or posttraumatic* or post-traumatic* or suicide* or suicidal* or (psycholog* adj3 (distress* or suffer* or condition* or disorder* or ill* or disease*))).ti,ab,kf.  7. ((substance* or drug*) adj2 (consumption* or use* or using or abus*)).ti,ab,kf.  8. 1 or 2 or 3 or 4 or 5 or 6 or 7  9. exp Health Services Accessibility/  10. (healthcare adj3 (access* or equit*)).ti,ab,kf.  11. (health adj2 (service* or care) adj2 (seek* or use* or using or utiliz* or access* or equit*)).ti,ab,kf. 12. Health card*.ti,ab,kf.  13. exp Insurance/  14. Insurance*.ti,ab,kf.  15. (mental health adj2 (seek* or access* or use* or using or utiliz* or service* or care)).ti,ab,kf.  16. ((use* or using or utiliz* or access* or seek*) adj3 (counsell* or psychotherap* or psycho-therap* or therap*)).ti,ab,kf.  17. help-seeking behavior/  18. (help adj2 seek*).ti,ab,kf.  19. 9 or 10 or 11 or 12 or 13 or 14 or 15 or 16 or 17 or 18  20. exp Students/  21. student*.ti,ab,kf.  22. 20 or 21  23. exp "Emigrants and Immigrants"/  24. "Emigration and Immigration"/  25. Refugees/  26. (immigra* or emigra* or refugee* or asylum seeker*).ti,ab,kf.  27. 23 or 24 or 25 or 26  28. 22 and 27  29. ((Exchange* or International* or permanent resident*) adj3 student*).ti,ab,kf.  30. ((Cultur* or linguisti*) adj2 divers* adj3 student*).ti,ab,kf.  31. 28 or 29 or 30  32. 8 and 19 and 31 |
| Study records: |  |  |
| Data management | 11a | To manage records and data throughout the review, we will use a bibliographic software, Zotero, to store, organize, and manage all references (39). Covidence will be used to manage the title/abstract and full-text screening phases. |
| Selection process | 11b | Two independent reviewers will review all articles in Covidence according to the above-mentioned inclusion/exclusion criteria. To increase consistency among reviewers, they will screen a sample of 20 publications, discuss the results and amended the screening and data extraction manual before beginning screening for this review. Two reviewers will be working in pairs sequentially to evaluate the titles, abstracts and then full text of all publications identified by the searches for potentially relevant publications. Disagreements on study selection and data extraction are expected to be solved by consensus and discussion with the third reviewer if needed. This will be done as follows: after importing the articles into Covalence, this software will automatically eliminate all duplicates (studies identified more than once by the search engines) and the two reviewers will start screening the articles. In a first step (first sort) the titles, abstracts and summaries (if any) will be examined and all studies that clearly do not meet the inclusion criteria will be excluded. Each study will be classified as "included" or "excluded" to identify relevant literature and to exclude irrelevant literature. If there is any doubt about the relevance of a publication, the reviewer may leave it for further evaluation to include or exclude it. In a second phase, all full-text articles that meet the inclusion criteria will be evaluated in more detail. All full-text articles will be carefully reviewed and those that do not meet the listed inclusion criteria will be excluded. At the end, we will only extract and analyze articles that met our inclusion criteria. |
| Data collection process | 11c | Data from articles that meet our inclusion criteria will be extracted into an Excel spreadsheet developed from the adaptation of the manual (data extraction) the Joanna Briggs Institute (14). This data extraction spreadsheet will be developed by a first reviewer who will be shared with a second reviewer (and a third reviewer if needed) to ensure that it will be reasonably interpreted and will capture all relevant data from different study designs to meet the objectives of the study. This Excel sheet will be developed and well-tailored to record key information from the studies to be analyzed. It will include authors, year of study, objective/purpose, geographic area (e.g., country), study population (e.g., age and gender of participants, degree on education), sample size, study design, results (e.g., barriers/facilitators) and key findings related to question and objective of this review. |
| Data items | 12 | None |
| Outcomes and prioritization | 13 | Barriers and facilitators associated with mental health services use by immigrant students in high income countries.  We will use a narrative review approach to collect and summarize the characteristics of each article (e.g., country of origin, type, and nature of the study, group of the age of participants, the date, results (types of barriers and facilitators of mental health services, etc.) and present them without attempting to assess the certainty of the results. |
| Risk of bias in individual studies | 14 | Appraisal for quality assessment or risk of bias will not be performed because this is a scoping review. |
| Data synthesis | 15a | We will involve quantitative (e.g., frequencies) and qualitative (e.g., content and thematic analysis) methods to present the data collected in different forms (tables, figures, texts). Synthesis of the results including socio-demographic characteristics factors and more elements of the participants will help in the analysis and consistency of this review. Graphs can also be used to present the results in a clearer and more understandable way. |
|  | 15b | Not applicable |
|  | 15c | Not applicable |
|  | 15d | Narrative synthesis |
| Meta-bias(es) | 16 | Language restriction: Relevant articles written in languages other than English may be missed because of limiting searches to only articles published in English language.  Inclusion of only primary studies: Because some secondary studies, such as systematic reviews, could include relevant articles on our topic. Since, we are not going to consider these types of studies, we may then miss these articles and not consider them. This could have an impact on the objectivity of our final analysis. |
| Confidence in cumulative evidence | 17 | Not applicable |

*** It is strongly recommended that this checklist be read in conjunction with the PRISMA-P Explanation and Elaboration (cite when available) for important clarification on the items. Amendments to a review protocol should be tracked and dated. The copyright for PRISMA-P (including checklist) is held by the PRISMA-P Group and is distributed under a Creative Commons Attribution Licence 4.0.**

*From: Shamseer L, Moher D, Clarke M, Ghersi D, Liberati A, Petticrew M, Shekelle P, Stewart L, PRISMA-P Group. Preferred reporting items for systematic review and meta-analysis protocols (PRISMA-P) 2015: elaboration and explanation. BMJ. 2015 Jan 2;349(jan02 1):g7647.*
