# Supplementary figures and images for: Barriers and facilitators associated with the use of mental health services among immigrant students in high-income countries: a scoping review protocol
Source: Syst Rev. 2022 Feb 6;11:22. doi: 10.1186/s13643-022-01896-6 (PMC8818184; doi:10.1186/s13643-022-01896-6)

**Draft of the data extraction form**

**
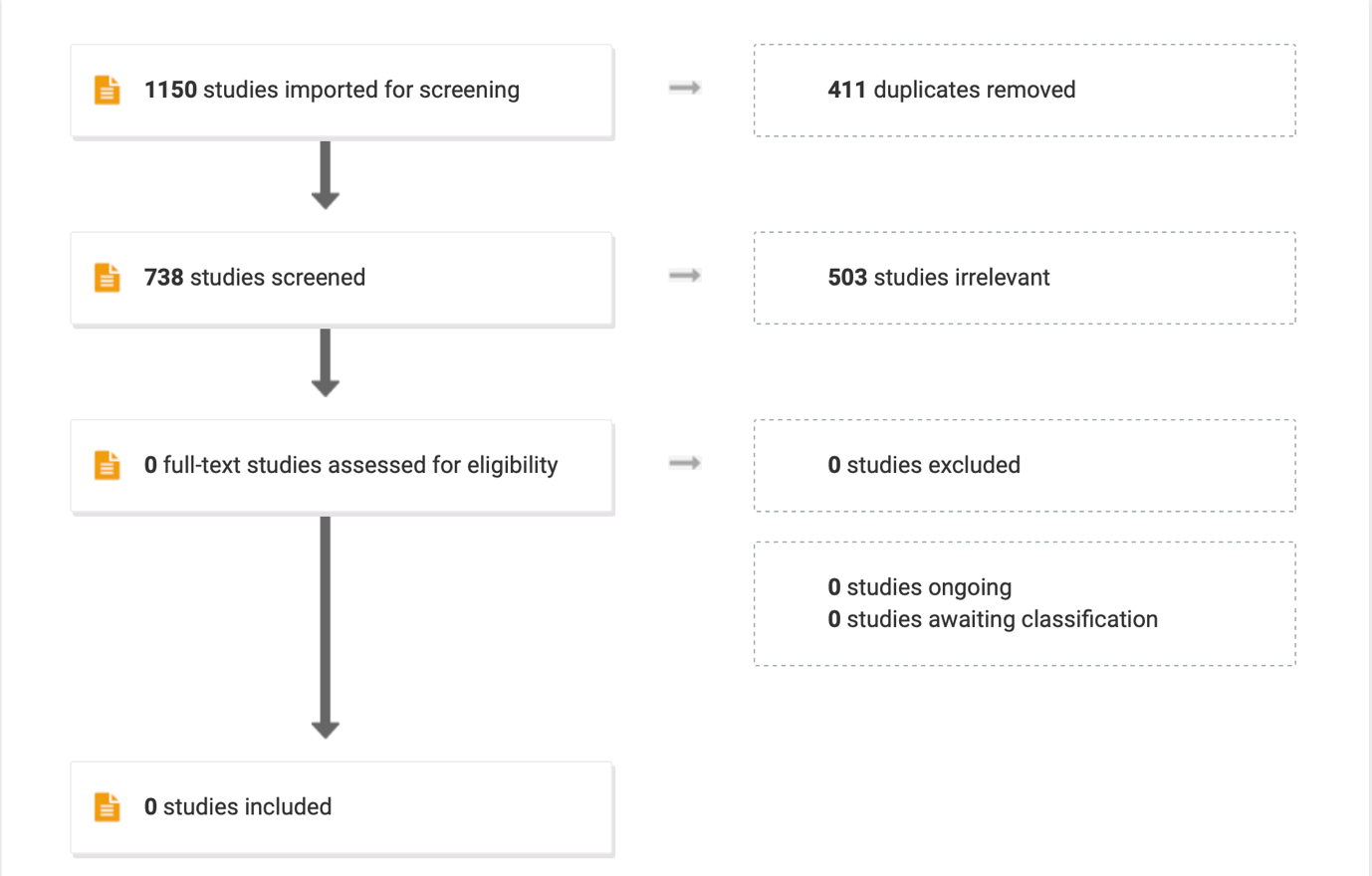
**

Supplement: Supplementary file 2 — Additional file 2. Draft search strategy for Medline. [file 13643_2022_1896_MOESM2_ESM.docx]
